# Patients with low activation level report limited possibilities to participate in cancer care

**DOI:** 10.1111/hex.13438

**Published:** 2022-01-20

**Authors:** Bodil Westman, Karin Bergkvist, Andreas Karlsson Rosenblad, Lena Sharp, Mia Bergenmar

**Affiliations:** ^1^ Regional Cancer Centre Stockholm‐Gotland Stockholm Sweden; ^2^ Department of Care Science Sophiahemmet University Stockholm Sweden; ^3^ Division of Nursing, Department of Neurobiology, Care Sciences and Society Karolinska Institutet Stockholm Sweden; ^4^ Division of Clinical Diabetology and Metabolism, Department of Medical Sciences Uppsala University Uppsala Sweden; ^5^ Division of Innovative Care Research LIME, Karolinska Institutet Stockholm Sweden; ^6^ Department of Oncology‐Pathology Karolinska Institutet Stockholm Sweden; ^7^ Department of Pelvic Cancer, Psychosocial Unit Karolinska University Hospital Stockholm Sweden

**Keywords:** cancer, patient activation, patient participation, person‐centred care, population‐based study

## Abstract

**Background:**

Cancer care trajectories are often complex, with potent multimodality treatments and multiple interactions with health care providers. Communication and coordination are challenging and the patients' responsibilities to take on more active roles in their own care are increasing.

**Objective:**

This study aimed to investigate associations between patient activation level and participation in cancer care, sociodemographic characteristics, clinical data, health‐related quality of life (HRQoL) and helpfulness of received information.

**Methods:**

In this cross‐sectional population‐based study, patients completed questionnaires on patient activation, perceived participation, HRQoL, helpfulness of received information and sociodemographic characteristics. Responses to the patient activation measures (PAMs) were classified into four levels (higher levels indicating more activation). Data on age, sex and cancer diagnosis were collected from the Swedish Cancer Register.

**Results:**

Data from 682 patients were analysed. On comparing patients at PAM levels 1 and 4, the latter reported significantly higher possibilities to influence care decisions (46.6% vs. 20.8%) and to ask questions regarding treatment and care (93.4% vs. 68.4%). Patients at PAM level 4 reported wanting to influence decision‐making to a higher extent, compared with patients at other PAM levels, and reported clinically significantly higher HRQoL. No significant differences were found regarding sociodemographic characteristics.

**Conclusion:**

We found strong associations between perceived patient participation and activation levels, with limited possibility for participation among those with lower activation levels.

**Patient or Public Contribution:**

Discussions with patient representatives have raised the importance of participation. The preliminary findings were presented and discussed in a workshop with representatives from 21 cancer patient advocacy groups.

## 1 INTRODUCTION

Patient participation has been the subject of several concept analyses.^1^, ^2^, ^3^ Cahill^2^ described a hierarchical relationship within the concept where involvement and collaboration were precursors for participation, with partnership as the goal. Collaboration and sharing of knowledge and power between patients and health care professionals (HCPs) were essentials to achieve partnership. Nilsson et al.^3^ identified learning, a caring relationship and reciprocity as the basis for patient participation and that a caring relationship promoted trust. Collaboration (described here as a relationship between patients and HCPs including sharing information, knowledge and power) was identified as vital for patient participation.^1^ Here the authors highlight the importance of the HCPs encouraging actions supporting patients’ knowledge and motivation to take active part in their care. Despite some differences in the descriptions of patient participation reflecting the lack of consensus, important commonalities are reported. Central and recurring topics include building relationships between the patient and the HCPs as well as information exchange for both parts to increase knowledge and understanding.

Research on patient participation has mainly concerned shared decision‐making related to treatment options.^4^, ^5^, ^6^, ^7^ It has, however, expanded to also include opportunities to acquire and apply knowledge about illness, treatment and survivorship.^8^, ^9^, ^10^, ^11^ Patients' opportunities to ask questions and HCPs' recognitions of patients' preferences have been identified as challenges in efforts aiming to improve patient participation.^4^, ^9^, ^10^ The importance of fulfilling information needs has been found to have an impact on health‐related quality of life (HRQoL).^12^ HRQoL is a broad concept covering a person's subjective perception of impact from illness. The concept measures, for example, physical, emotional, cognitive and social aspects as well as symptoms of disease, treatment side effects and well‐being.^13^, ^14^

Cancer patients' care trajectories are often complex. More often, treatments are administered at outpatient clinics, primary care or even in the patients' home. As a consequence, the responsibility for patients to coordinate their care and self‐manage side‐effects has increased.^15^, ^16^ Patients are therefore expected to take on a more active role in their own care. Patient activation has been defined as having the knowledge, skills and the confidence to take on an active role in one's own care, to self‐manage symptoms and collaborate with HCPs to maintain health.^17^ Patients with high activation levels are more likely to have improved health outcomes and more positive care experiences.^18^, ^19^ Associations between low levels of patient activation and being discouraged and overwhelmed when managing health issues have been found.^20^ Low levels of activation have also been associated with less care satisfaction and poorer understanding of one's diagnosis.^21^ However, knowledge of patients' different activation levels and possibilities to participate in their own care is sparse. Complex treatments and fragmented care have also been identified as barriers to participation.^22^ Patients diagnosed with gynaecological, haematological, head and neck (H&N) or upper gastrointestinal (GI) cancer usually undergo complex treatments. Few studies have been carried out regarding patient participation and activation among these groups.^23^, ^24^ Therefore, more knowledge regarding factors influencing patients' possibilities to participate in their own care is of interest. The present study examines the associations between cancer patients' perceptions of participation and their activation level in a Swedish context.

### 1.1 Aim

The primary aim was to investigate the associations between patient‐reported participation in cancer care and patient activation level. The secondary aims were to investigate how sociodemographic characteristics, clinical data as well as HRQoL and helpfulness of received information were associated with patient activation.

## 2 METHODS

In this cross‐sectional, population‐based study, patient‐reported and registry data were collected. The study was approved by the Swedish Ethical Review Authority (Dnr 2019‐04582).

### 2.1 Inclusion criteria

All patients aged ≥18 years diagnosed during 2018 with gynaecological, haematological, H&N or GI cancer in the Stockholm–Gotland region, Sweden, were invited to participate in the study.

### 2.2 Data collection

The patients were identified through the Swedish Cancer Register, which includes 99% of all clinically and morphologically reported cancer cases in Sweden.^25^ To minimize the risks of sending the questionnaires to deceased persons, data on cancer diagnosis were linked to the National Population Register using each individual's unique Swedish personal identification number. The invitation to participate was sent by regular post, together with the questionnaires (described below) and an information letter, to potential participants during November and December 2019, a time period when most patients had completed their primary treatment. The letter described the purpose of the study, that participation was voluntary, the confidentiality process and that a completed questionnaire was considered consent to participate. One reminder was sent to those not responding within 3 weeks. The questionnaires could be filled in using paper and pen, for which a prestamped envelope was included, or online via a secure internet link. Contact information to the responsible researcher was included, giving potential participants possibilities to ask questions about the study. In addition, contact information to a nurse‐led regional cancer support service was included.

### 2.3 Questionnaires

For the purpose of this study, we used the following questionnaires (presented below) to collect data on patient activation, HRQoL, helpfulness of received information, perceived participation and sociodemographic characteristics.

#### 2.3.1 Patient activation

The Patient Activation Measure (PAM‐13®) scale was developed to measure a person's knowledge, skills, beliefs and how comfortable they felt taking on an active role in their care.^26^ The PAM‐13® scale has been widely used for measuring patient activation in different populations, including patients with cancer,^21^, ^27^, ^28^ and is validated in Swedish.^29^ The instrument consists of 13 items with 4‐point response options: ‘disagree strongly’, ‘disagree’, ‘agree’ and ‘agree strongly’, as well as a ‘not applicable’ option. Scores are weighted and transformed into a 0–100 scale and thereafter converted into four ordinal levels: PAM 1, ‘Disengaged and overwhelmed’; PAM 2, ‘Becoming aware but still struggling’; PAM 3, ‘Taking action and gaining control’; and PAM 4, ‘Maintaining behaviours and pushing further’. Higher levels thus indicate higher knowledge, skills and confidence.^26^

#### 2.3.2 HRQoL and information

The HRQoL core questionnaire (EORTC QLQ‐C30) developed by the European Organization for Research and Treatment of Cancer (EORTC) consists of 30 items, including a global health status/QoL scale, five functional scales (physical, role, emotional, social, cognitive), three symptom scales and six single‐item scales. In this study, the functional scales and the global health status/QoL scale were analysed. A 4‐point response scale ranging from 1 (*not at all*) to 4 (*very much*) was used for all, except the items pertaining to global health status/QoL, which ranged from 1 (*very poor*) to 7 (*excellent*).

Patient‐reported data on information related to cancer treatment and care were collected using the information module EORTC QLQ‐INFO25,^30^ an add‐on module to the EORTC QLQ‐C30 questionnaire. It consists of 25 items organized into four multiitem scales: information about the disease, medical tests, treatment and other services, and eight single‐item scales. For this study, the item ‘overall helpfulness of information’ was analysed. Both instruments have been developed and validated within the cancer context.^30^, ^31^

#### 2.3.3 Patient participation

Data on patients' perceptions of their own participation within the cancer care context were collected using a locally developed questionnaire, which had been used in previous studies.^22^, ^32^ The present study analysed six questions concerning patient participation and six sociodemographic questions. The patient participation questions (Q1–Q6) are presented in Table 1. The response options for Q1–Q5 were ‘not at all’, ‘a little’, ‘a lot’ or ‘very much’, while Q6 was answered on a 3‐point scale (no; yes, to some extent; yes, absolutely).

**Table 1:** Patient participation in cancer care, *n* (%), in relation to PAM level for the 682 participants

Question	PAM level				*p* value^a^	Missing
	1	2	3	4		*n* (%)
Q1. Did you have the possibility to influence decisions regarding your treatment?^b^	15 (20.8)	52 (30.1)	80 (34.3)	82 (46.6)	**<0.001**	28 (4.1)
Q2. Did you wish to have more influence regarding decision‐making related to your treatment?^c^	42 (58.3)	110 (62.9)	156 (66.4)	137 (77.0)	**0.008**	22 (3.2)
Q3. Did you feel comfortable raising your opinions regarding your care?^b^	38 (52.1)	119 (72.1)	179 (77.5)	155 (86.1)	**<0.001**	33 (4.8)
Q4. Did you have the possibility to ask questions regarding your care and treatment if there was something you didn't understand?^b^	52 (68.4)	144 (83.2)	208 (88.1)	169 (93.4)	**<0.001**	16 (2.3)
Q5. Did the staff take your wishes into account when planning your care, for example, current times for examinations and treatments?^b^	35 (47.3)	116 (70.3)	170 (73.9)	147 (80.8)	**<0.001**	31 (4.5)
Q6. Have you been involved to the extent you wanted in the decisions about your care and treatment?^d^	56 (73.7)	150 (87.7)	212 (89.5)	170 (94.4)	**<0.001**	18 (2.6)

*Note*: Significant *p* values are given in bold.

Abbreviation: PAM, patient activation measure.

^a^ *p* Values calculated using Pearson's *χ* ^2^ test.

^b^ Patients answering ‘quite a lot’ or ‘a lot’ were considered to be participating in his/her cancer care.

^c^ Patients answering ‘not at all’ were considered to be participating in his/her cancer care.

^d^ Patients answering ‘yes, to some extent’ or ‘yes, absolutely’ were considered to be participating in his/her cancer care.

### 2.4 Statistical analyses

Response scores for the EORTC QLQ‐C30 functional scales, the global health status/QoL and EORTC QLQ‐INFO25 were linearly transformed into 0–100 scales, according to the EORTC manual, where higher scores represent better functioning and global health status/QoL (EORTC QLQ‐C30) and higher rating of the information received (EORTC‐QLQ‐INFO25), respectively.^33^ The mean scores were calculated for each scale.^33^ Differences in scale scores were considered clinically significant according to the thresholds for clinically significant changes reported in Osoba et al.,^34^ where changes of 5–10 points on a 0–100 scale were considered as ‘small’, 10–20 as ‘moderate’ and changes of 20 points or more were considered as a ‘large’ change.

According to the PAM‐13® manual, the continuous PAM score is intended for tracking changes in activation level over time, while PAM levels can provide information about the individual patient's capacity for self‐management at a specific time. For the present study, it was decided to use the PAM level for assessing associations with perceptions of patient participation.

Categorical data are presented as frequencies and percentages, *n* (%), while ordinal and continuous data are given as means with accompanying standard deviations. Tests of differences between categorical variables were calculated using Pearson's *χ* ^2^ test. In the univariate analyses of patient participation (yes/no), patients answering ‘very much’ or ‘a lot’ were considered to be participating in their cancer care and were categorized as ‘yes’. For question Q2 the response “not at all” were categorised as “yes” and for question Q6 the responses “yes, to some extent” and “yes, absolutely” were categorised as “yes”. Unadjusted and adjusted logistic regression models were used to estimate the magnitude of the effect of patient participation and sociodemographic factors on patient activation, reported as odds ratios with accompanying 95% confidence intervals (CIs). PAM‐13® levels 3 and 4 were defined as being active, while Levels 1 and 2 were defined as being inactive (reference category). The responses ‘a lot’ and ‘very much’ for the participation questions were merged into one category. ‘Not at all’ was used as the reference category in the logistic regression models, except for Q2, which used ‘a lot/very much’ as a reference category. Age (years) was included as a continuous variable in the logistic regression models, while male sex, foreign born, cohabiting and college/university education were included as categorical variables (yes/no), using ‘no’ as the reference category. In the adjusted models, cancer type was included as an adjustment variable, with upper GI cancer used as the reference category. All regression models used a complete cases analysis approach, i.e. no imputations were used. The le Cessie–van Houwelingen–Copas–Hosmer test^35^ was used for examining the global goodness of fit. Statistical analyses were performed using *R* ≥ 4.0.0 (R Foundation for Statistical Computing), with two‐sided *p* values less than .05 considered statistically significant.

## 3 RESULTS

### 3.1 Patient characteristics

**Figure 1:**
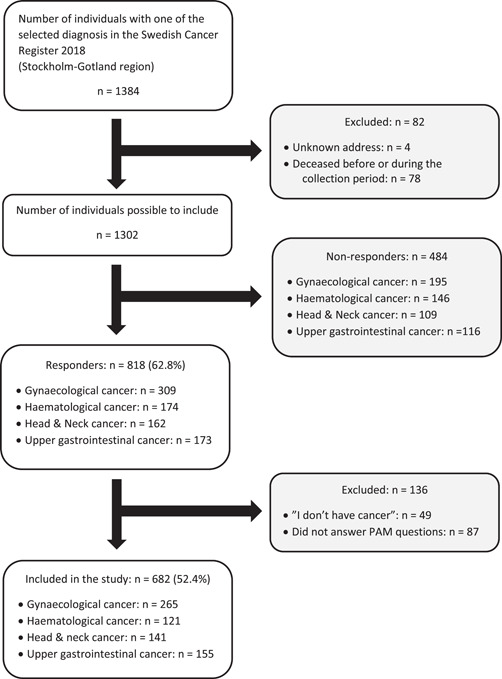
PRISMA Flowchart

Out of 1302 patients who fulfilled the inclusion criteria, 818 (62.8%) responded to at least one of the questionnaires, while 682 patients (52.4%) completed the PAM‐13® scale (Figure 1). No significant differences were found between the 818 responders and the remaining 566 nonresponders and excluded individuals regarding sex (*p* = .879) or cancer type (*p* = .251), but the two groups differed in age at survey allocation (*p* < .001), with the responders (mean age: 67.8 years) on average being 3.3 years older. The characteristics of the 682 patients included in the study are presented in Table 2. Most patients were women (62.9%; *n* = 429), born in Sweden (81.8%; *n* = 558), cohabiting (62.7%; *n* = 421), did not have a college or university degree (57.1%; *n* = 396) and were retired (66.6%; *n* = 450). Gynaecological cancer was the most common cancer type (38.9%; *n* = 265). The majority (56.1%; *n* = 370) of the patients reported that they had received single‐modality cancer treatment and one‐third (34.0%; *n* = 222) reported having been referred to palliative care.

**Table 2:** Background characteristics of the 682 participants according to PAM levels

Characteristic	Variable	PAM 1 (*n* = 78)	PAM 2 (*n* = 176)	PAM 3 (*n* = 242)	PAM 4 (*n* = 186)	*p* Value	Total (*n* = 682)	Missing, *n* (%)
Demographic	Age (years), mean (SD)^a^	68.4 (13.2)	67.7 (13.6)	66.7 (13.2)	66.5 (12.1)	.328	67.1 (13.0)	0 (0.0)^b^
	Age group, *n* (%)					.814		0 (0.0)^b^
	<50 years	9 (11.5)	21 (11.9)	30 (12.4)	19 (10.2)		79 (11.6)	
	50–<65 years	15 (19.2)	41 (23.3)	51 (21.1)	50 (26.9)		157 (23.0)	
	≥65 years	54 (69.2)	114 (64.8)	161 (66.5)	117 (62.9)		446 (65.4)	
	Male sex, *n* (%)	30 (38.5)	55 (31.2)	106 (43.8)	62 (33.3)	**.038**	253 (37.1)	0 (0.0)^b^
	Foreign born, *n* (%)	17 (21.8)	23 (13.1)	53 (21.9)	31 (16.7)	.097	124 (18.2)	0 (0.0)^b^
	Cohabiting, *n* (%)	45 (57.7)	108 (61.4)	145 (61.7)	123 (67.6)	.403	421 (62.7)	11 (1.6)
	College/university education, *n* (%)	30 (38.5)	66 (37.7)	105 (45.3)	85 (46.7)	.246	286 (42.9)	15 (2.2)
	Retired, *n* (%)^c^	55 (70.5)	116 (67.1)	159 (65.7)	120 (65.6)	.867	450 (66.6)	6 (0.9)
Clinical	Cancer type, *n* (%)					.382		0 (0.0)^b^
	Gynaecological	29 (37.2)	69 (39.2)	81 (33.5)	86 (46.2)		265 (38.9)	
	Upper gastrointestinal	20 (25.6)	37 (21.0)	63 (26.0)	35 (18.8)		155 (22.7)	
	Head & neck	16 (20.5)	39 (22.2)	55 (22.7)	31 (16.7)		141 (20.7)	
	Haematological	13 (16.7)	31 (17.6)	43 (17.8)	34 (18.3)		121 (17.7)	
	Received single‐modality treatment, *n* (%)	41 (55.4)	106 (61.6)	116 (49.4)	107 (59.8)	.059	370 (56.1)	22 (3.2)
	Received palliative care referral, *n* (%)	32 (42.1)	56 (33.3)	92 (39.8)	42 (23.6)	**.002**	222 (34.0)	29 (4.3)
	Access to rehabilitation contacts, *n* (%)^c^	53 (68.8)	110 (63.6)	158 (66.7)	104 (57.1)	.161	425 (63.5)	13 (1.9)
Quality of life^d^	Global health status/QoL (0–100 points), mean (SD)	47.8 (23.4)	64.3 (21.8)	64.7 (20.1)	77.7 (18.9)	**<.001**	66.2 (22.4)	13 (1.9)
	Physical functioning (0–100 points), mean (SD)	62.2 (27.8)	78.0 (23.2)	78.8 (21.2)	87.1 (16.3)	**<.001**	79.0 (22.5)	11 (1.6)
	Role functioning (0–100 points), mean (SD)	57.9 (34.9)	75.5 (30.1)	72.2 (32.6)	87.1 (24.3)	**<.001**	75.5 (31.3)	14 (2.1)
	Emotional functioning (0–100 points), mean (SD)	62.2 (25.6)	77.3 (23.5)	75.1 (25.0)	84.8 (19.3)	**<.001**	76.9 (24.1)	13 (1.9)
	Cognitive functioning (0–100 points), mean (SD)	71.1 (24.4)	83.7 (19.3)	79.3 (21.9)	89.2 (17.5)	**<.001**	82.2 (21.2)	13 (1.9)
	Social functioning (0–100 points), mean (SD)	55.9 (32.3)	77.2 (27.7)	75.0 (29.0)	89.5 (19.7)	**<.001**	77.4 (28.5)	15 (2.2)

*Note*: Significant *p* values are given in bold.

Abbreviations: PAM, patient activation measure; QoL, quality of life; SD, standard deviation.

^a^ Min: 23.8 years, max: 93.8 years.

^b^ Register‐based data.

^c^ Multiple choices possible.

^d^ Measured by EORTC QLQ‐C30.

Overall, 11% (*n* = 78) of the patients were identified as PAM level 1 (i.e., representing low levels of activation), 26% (*n* = 176) as PAM level 2, 35% (*n* = 242) as PAM level 3 and 27% (*n* = 186) as PAM level 4 (i.e., representing high levels of activation), (Table 2). Significant differences were found between patients in the different PAM levels regarding gender, with 43.8% (*n* = 106) men at PAM level 3 compared to 31.2% (*n* = 55) at PAM level 2. Referral to palliative care differed significantly, with 42.1% (*n* = 32) of patients at PAM level 1 reporting that they had received palliative care referral compared to 23.6% (*n* = 42) at PAM level 4. Patients in the PAM 4 group scored the highest on HRQoL (measured by EORTC QLQ C‐30). Both the global health status/QoL scale and the five functional scales (physical, role, emotional, social, cognitive) differed significantly between PAM levels, with the observed scores for both the role and social function as well as global health status/QoL being clinically significantly higher for patients at PAM level 4 compared to the other PAM levels (Table 2).

### 3.2 Perceived participation

In univariate analyses, the questions regarding patient participation (Q1–Q5 from the locally developed questionnaire) in cancer care (yes/no) differed significantly between PAM levels for all questions (Table 3). After adjusting for patient characteristics, those who reported that they ‘felt more comfortable to raise opinions regarding their care’ (Q3) or ‘staff taking your wishes into account when planning your care’ (Q5) were, respectively, 2.402 (95% CI: 1.098–5.343) and 1.970 (95% CI: 1.109–3.513) times more likely to be classified as active patients (PAM level 3 or 4).

**Table 3:** Results from unadjusted and adjusted logistic regression analyses of predictors of patient activation for the 682 participants

Variable	Unadjusted	*p* Value	Adjusted^a^	*p* Value
	OR (95% CI)		OR (95% CI)	
Age (years)	0.992 (0.980–1.004)	.201	0.996 (0.982–1.010)	.562
Male gender	1.285 (0.930–1.782)	.131	1.438 (0.898–2.303)	.130
Foreign born	1.306 (0.869–1.991)	.205	1.255 (0.789–2.025)	.344
Cohabiting	1.187 (0.860–1.637)	.295	0.965 (0.663–1.397)	.850
College/university education	1.387 (1.009–1.912)	.044	1.394 (0.967–2.017)	.076
Cancer type				
Upper gastrointestinal	Ref.		Ref.	
Gynaecological	0.991 (0.656–1.493)	.966	1.387 (0.813–2.359)	.228
Head & neck	0.909 (0.568–1.456)	.692	1.211 (0.711–2.070)	.481
Haematological	1.018 (0.622–1.671)	.944	1.156 (0.650–2.071)	.622
Q1. Did you have the possibility to influence decisions regarding your treatment?				
Not at all	Ref.		Ref.	
A little	1.007 (0.683–1.482)	.974	0.762 (0.475–1.213)	.255
A lot/very	1.749 (1.170–2.623)	**.007**	0.987 (0.586–1.650)	.960
Q2. Did you wish to have more influence regarding decision‐making related to your treatment?				
A lot/very	Ref.		Ref.	
A little	1.259 (0.713–2.222)	.426	0.945 (0.484–1.829)	.866
Not at all	1.775 (1.075–2.927)	**.024**	1.058 (0.550–2.014)	.864
Q3. Did you feel comfortable raising your opinions regarding your care?				
Not at all	Ref.		Ref.	
A little	1.809 (0.937–3.542)	.080	1.989 (0.887–4.547)	.098
A lot/very	3.288 (1.874–5.878)	**<.001**	2.402 (1.098–5.343)	**.029**
Q4. Did you have the possibility to ask questions regarding your care and treatment if there was something you didn't understand?				
Not at all	Ref.		Ref.	
A little	2.357 (0.735–9.091)	.171	1.693 (0.396–9.011)	.497
A lot/very	5.290 (1.783–19.283)	**.005**	1.976 (0.476–10.264)	.371
Q5. Did the staff take your wishes into account when planning your care, for example, current times for examinations and treatments?				
Not at all	Ref.		Ref.	
A little	1.714 (0.951–3.115)	.074	1.789 (0.925–3.494)	.085
A lot/very	2.655 (1.630–4.355)	**<.001**	1.970 (1.109– 3.513)	**.021**
Q6. Have you been involved to the extent you wanted in the decisions about your care and treatment?				
No	Ref.		Ref.	
Yes, to some extent	1.518 (0.894–2.587)	.123	0.945 (0.467–1.888)	.873
Yes, absolutely	2.661 (1.615–4.406)	**<.001**	1.306 (0.630–2.673)	.468

*Note*: Significant *p* values are given in bold. Patient activation is defined as answers on the PAM questionnaire resulting in Levels 3 and 4, with Levels 1 and 2 used as the reference.

Abbreviations: CI, confidence interval; OR, odds ratio; Ref., reference category.

^a^ Adjusted for all other variables in the table. Results based on 594 observations with complete cases. The *p* value for the le Cessie–van Houwelingen–Copas–Hosmer global goodness‐of‐fit test was 0.856.

### 3.3 Perceived information

**Figure 2:**
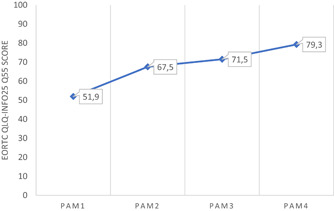
Mean values for EORTC QLQ‐INFO25 and Q55 (the overall information was helpful) in relation to PAM level for the 682 participants. PAM, patient activation measure

Patients at PAM level 4 scored higher (79.3 points) on the EORTC‐INFO25 item of whether ‘the overall information was helpful’ compared to patients at PAM level 1 (51.9 points; Figure 2). The differences in the mean scores for ‘the overall helpfulness of the information’ corresponded to clinically significant differences between all PAM levels, except between PAM levels 2 and 3.

## 4 DISCUSSION AND CONCLUSION

### 4.1 Discussion

In this population‐based study among patients treated for cancer, strong associations were found between perceived patient participation and activation level, with more reported participation among those with higher activation levels. Patients at PAM level 4 reported greater possibilities to influence decisions and to ask clarifying questions, feeling more comfortable raising opinions and finding the received information more helpful. Patients at PAM level 1 reported to a higher degree of not being involved in their care and treatment decisions to the extent that they had preferred. Patients at PAM level 1 also reported lower HRQoL on all functional scales and also on overall global health status/QoL compared with patients at PAM level 4. The HRQoL scores for patients at PAM level 4 were comparable with norm data based on the Swedish general population stratified for sex and age.^36^ The scores, reported by patients at PAM level 1, for both the physical role and social functional scales as well as global health status/QoL were more than 20 points lower compared to the general population, a difference that corresponds to a ‘large’ clinically significant change.^34^ For the emotional and cognitive functional scales, the differences in scores were 10–20 points, corresponding to a ‘moderate change’. Patients at PAM level 1 also received referral to palliative care and had access to rehabilitation contacts to a higher extent compared with patients at PAM level 4. This might reflect poorer health among patients at PAM level 1, which in turn may impact their ability to take on an active role in their care.

A higher proportion of patients at PAM 1 reported a preference for being more involved in decisions regarding treatment and care, compared to patients at PAM 4. Discrepancies between cancer patients' preferred and actual involvement in decision‐making have previously been found in a German study, which reported the majority of patients (75%) wanting to have a more shared and active role in treatment decisions than experienced.^37^ In another study among patients with GI cancer, 46% reported involvement in decision‐making related to care and treatment to the extent they preferred.^38^ In the present study, 58% of patients at PAM level 1 and 77% of patients at PAM level 4 reported that they would have preferred more influence related to treatment options. This could be compared with a previous study, including patients with the same diagnostic groups in the same geographical area, where the corresponding proportion was only 10%.^22^ Moreover, the present study found some discrepancies, with patients wanting more influence in decision‐making related to their treatment and wishing to be more involved in decisions regarding care and treatment, regardless of the PAM level. In the locally developed questionnaire, some items combined *care* and *treatment* in the same question; thus, it was impossible to differentiate if responses were to the question of care or treatment, respectively. Patients might have felt that they had more possibilities to influence decisions regarding their care, rather than regarding treatment decisions.

Both patients' and HCPs' attitudes and knowledge have been found to impact patient participation. In a recent review, Halabi et al.^1^ concluded that patient participation requires HCPs to shift from ‘doing to’ to ‘working with’ patients. This includes sharing information, knowledge and power. Data on patients' views of HCPs' attitudes related to patient participation were not collected in the present study. However, many of the responders with lower activation levels reported feeling uncomfortable in expressing opinions regarding their care (52.1%) and limited possibilities to ask questions (68.4%). These results indicate that the attitudes/actions by the HCPs may have had an impact, failing to identify the individual needs of adapted information. Patients with high activation levels have, in a previous study, been identified as more likely to be active in searching information and asking questions, thereby being better informed.^21^ Having the possibility to receive responses to questions and additional explanations when not understanding the information were important for patients undergoing cancer treatments and vital for feeling respected as a person. It also affected patients' confidence and trust in care.^39^

Being informed and having knowledge have been described as fundamental and prerequisites for patient participation in previous research. In the present study, the mean score for patients at PAM level 1 reporting that the information overall had been helpful was 51.9, compared to 79.3 for patients at PAM level 4. This may reflect feelings of being overwhelmed with too much information, which characterizes patients at PAM level 1,^21^ but it may also reflect scanty information or insufficient adaption of information to the individual's needs. Previous research, including patients with upper GI cancers,^38^, ^40^ found that individualized information, possibilities to ask questions and an ability to express personal views regarding one's own situation stimulated patient involvement.

The PAM‐13® scale was developed by Hibbard et al.^17^, ^26^ and aimed to measure patients' activation levels so that strategies could be tailored to support patients at the different levels to increase their activation by gaining knowledge, skills and confidence in making informed choices.^41^ Given the current situation with growing demands on patients to manage symptoms and side‐effects from complex treatments with less supervision from HCPs, the patient's ability to self‐management is important. Previous research has shown that activation levels impacted cancer patients' abilities to cope with side effects from treatment as well as the probability of patients following treatment regimens and making lifestyle changes that improved health outcomes^19^, ^41^ and reduced costs.^18^, ^19^

It is, however, important to bear in mind that not all patients want to take on an active role or have the prerequisites to take responsibility for their care.^42^ Identifying each patient's preferences regarding the extent to which he or she wants to participate in his or her own care is central in person‐centred care.^43^ Patients may have a desire to be informed, but not wishing to actively participate in decision‐making.^44^, ^45^ This can be related to collaboration, described by Cahill^2^ as the lowest level of participation. Still a main issue, reported by patients in the present study, was that patients did not have the opportunity to be involved to the extent they wished. To be able to improve the care of individual patients, it is important to promote active patient participation by improving communication and information exchange between HCPs and patients.^1^, ^10^, ^11^ Bol et al.^44^ described three profiles of information needs indicating the complexity and heterogeneity of information needs among patients with cancer. The ‘information seeker’ was characterized by high information need, while the ‘listener’ had a lower and less specific information need and finally, the ‘information avoider’ was reported to have a low information need, was less engaged and motivated and had lower self‐confidence in interactions with HCPs. Interestingly, and importantly, the authors found no significant differences regarding preferences for participation between these three profiles.^44^ HCPs have an ethical and legal obligation to inform patients about their treatment and care and also to reassure that the information is understood. Therefore, HCPs ought to adapt the information to the individual prerequisites. When measuring patients' activation level, HCPs still must identify and respect the individual ability and right to access adapted support and those with low activation levels should be given most support.^42^, ^46^

#### 4.1.1 Strengths and limitations

The present large population‐based study used the Swedish Cancer Register to identify presumptive participants, thus minimizing the risk for selection bias. It also gave all patients with a cancer diagnosis and understanding Swedish in the Stockholm–Gotland region equal possibilities to participate. Two validated questionnaires and additional nonvalidated study‐specific questions were used to collect data. The study had a response rate comparable with previous studies of the same diagnostic groups.^22^, ^47^ Although the survey was available only in Swedish, the percentage of responders born outside Sweden could be considered as representative of the population in the Stockholm region during 2018, where 25% of inhabitants >55 years old were born outside Sweden.^48^ A limitation of the results for the adjusted logistic regression analysis was that there were only 594 observations with complete cases out of a total of 682 participants, resulting in a total of 12.9% missing observations for this analysis.

The study contributes with new knowledge of patient activation and participation in the included groups and we see no reason why the results cannot be generalized to patients with other cancer types as well. The individual patient's perceptions of information received and perceived participation in his or her own care are probably not dependent on specific cancer diseases. There is no reason to believe that a certain diagnosis per se entails specific information needs. This question is probably more related to fragmented care, complexity in treatment regimens, personal circumstances and the HCPs' ability to individualize the information.

### 4.2 Conclusions

We found strong associations between perceived patient participation and activation levels, with more reported participation among those with higher activation levels. The fact that patients at PAM level 1 reported not being involved to the extent they wanted indicates the importance of systematically identifying the patients with the highest need for support and tailoring interventions to better meet these needs.

### 4.3 Practice implications

Advances in cancer treatment and care with improvements in survival but also a shift towards shorter hospital stays have led to an increased importance of patients' self‐management. It is therefore of great importance to identify patients with a low activation level to support them in gaining the knowledge and confidence needed to be more involved and take on an active role in their own care and self‐management. Measuring PAM in clinical settings could be a facilitator for HCP to identify patients who need support regarding ability, motivation and confidence in clinical encounters and foster individual support. Knowledge of the patient's PAM level could improve the HCPs' possibilities to adapt their communication accordingly. More research is needed to understand how patients express their preferred participation as well as research about how HCPs are aware of and responsive to these expressions.

## CONFLICT OF INTERESTS

The authors declare that there are no conflict of interests.

## AUTHOR CONTRIBUTIONS

**Bodil Westman**: Conceptualisation; Methodology; Investigation; Writing – original draft, project administration. **Karin Bergkvist**: Conceptualisation; Methodology; Writing – review and editing. **Andreas Karlsson Rosenblad**: Methodology; Software; Formal analysis; Data curation, Writing – review and editing. **Lena Sharp**: Conceptualisation; Methodology; Writing – review and editing. **Mia Bergenmar**: Conceptualisation; Methodology; Writing – review and editing. Supervision.

## Data Availability

Data are available upon reasonable request. Ethical restrictions related to participant confidentiality prohibit the authors from making the data set publicly available. During the consent process, participants were explicitly guaranteed that the data would only be seen by members of the study team. For any discussions about the data set, please contact the corresponding author.
